# Patella component diameters of 38 mm and up might be associated with higher revision rates after patella resurfacing

**DOI:** 10.1186/s13018-023-03705-9

**Published:** 2023-04-28

**Authors:** Anna Jungwirth-Weinberger, Kilian List, Ulrich Bechler, Carola Hanreich, Stefan Rueckl, Friedrich Boettner

**Affiliations:** 1 Hospital for Special Surgery, New York, USA; 2 Evangelisches Krankenhaus, Wien, Austria; 3 Orthopädische Klinik König-Ludwig-Haus, Würzburg, Germany; 4 Universitätsklinik Hamburg-Eppendorf, Hamburg, Germany; 5 Inselspital, Bern, Switzerland

**Keywords:** Patella resurfacing, Patella loosening, Total knee arthroplasty, Patella

## Abstract

**Purpose:**

Patellar resurfacing is considered the standard of care for total knee arthroplasty in the USA. Complications of patella resurfacing include aseptic loosening or patella fractures and can threaten the integrity of the extensor mechanism. The goal of this study was to report on patella button revision rates in posterior stabilized total knee arthroplasty.

**Material and methods:**

Between 01/2010 and 08/2016 patella buttons were implanted in 1056 patients (267 men and 550 women) as part of a posterior stabilized total knee arthroplasty.

**Results:**

Of 1056 cases, 35 cases (14 women, 15 men, 5 bilateral, 3.3%) showed early loosening at a mean 52.5 months postoperatively. Patella components of 38 mm or larger diameters showed a significantly higher loosening rate than the 29, 32, 35 mm buttons (*p* < 0.01). Mean BMI of patients identified with aseptic loosening was 31.7 kg/m^2^, mean age at time of revision surgery was 63.3 years. All of the patients with loosening of the patella button required revision surgery; in 33 cases an exchange of the button was performed, in two cases a removal of the button and patellar bone grafting was indicated. No complications occurred after revision surgery.

**Conclusion:**

The current study reports a 3.3% patella loosening rate during this mid-term follow-up. Size 38 mm and larger patella components showed a significantly higher revision rate than smaller buttons and the authors advise caution when using large diameter patella components.

## Introduction

The implantation of a total knee arthroplasty (TKA) has been shown to be a safe option for the treatment of advanced osteoarthritis of the knee. Initial implants did not include patella resurfacing. In the past, uncemented metal-backed patella components resulted in high complication rates [1], and cemented all-polyethylene patella components ultimately became the gold standard [2, 3]. Recent studies show lower revision rates when using patella buttons compared to non-resurfacing [4], and a large study of 11,887 TKAs observed that 92% of patella-resurfaced TKA were not revised after 15 years compared to 91% in non-patella resurfaced TKA [5]. According to the American Joint Replacement Registry TKAs with a resurfaced patella had a higher survivorship than cases without patella resurfacing, although the numbers were considerably higher in the resurfaced group (> 88,000 vs > 4000 cases) [6].

The Swedish joint registry demonstrates an exchange of the patella in primary TKA in 0.3% [7].

The goal of patella resurfacing is to reduce the risk for postoperative anterior knee pain [8] and improve knee function in flexion.

Aseptic loosening of patella component can compromise the patella bone stock and can threaten the integrity of the extensor mechanism, requiring the use of patella bone grafting or extensor mechanism allograft reconstructions.

The Genesis II TKR (Smith&Nephew, Memphis, TN) consists of a round 7.5 mm thick all polyethylene 29, 32 and 35 mm patella button as well as a 9 mm thick 38 and 41 mm patella button. The implant was introduced in 1996 and showed a low complication rate and a 10-year survival of 96% ± 2% [9] and 15-year survival rate of 96.4% (95.5–97.3%) [10].

The loosening of a patella component occurs in 0.4–9.5% [11–14] of patients according to the literature. One risk factor for loosening of a patella component or patella fractures is a body mass index (BMI) of greater than 30 kg/m^2^. Additional risk factors for patella loosening are a history of a lateral release, which leads to avascular necrosis of the patella, preoperative Valgus alignment of more than ten degrees, preoperative flexion of more than 100 degrees and thickness of the tibial component of more than 12 mm [12, 15]; medial positioning of the patella button reduces peak lateral shear forces [16]. Femoral component malposition has an impact on patellofemoral tracking as well as the risk of aseptic loosening, anterior knee pain, patella fracture and patella wear. Internal rotation of the femoral component was found to be the greatest risk factor for patellar failure [17, 18].

The purpose of this study is to report the single surgeon outcome data for the use of the Genesis II patella button in TKA and to report clinical and radiological outcome, survival and complication rate. The hypothesis of the study is that there is no difference in loosening rates of patella buttons of different sizes and diameters.

## Material and methods

This retrospective, comparative study was approved by the institutional review board (IRB) at the authors’ institution. Written informed consent was waived, as all data were retrospectively collected from patients' charts.

Between January 2010 and August 2016, a consecutive series of 1056 TKA were implanted by the senior author in 167 males and 550 females using the same technique in all cases. Patella buttons were used routinely in every TKA.

Inclusion criteria for this study were all patients undergoing primary TKAs operated by the senior author. All operations were performed under spinal-epidural anesthesia using a tourniquet.

During surgery, a medial parapatellar approach was performed and the patella was everted. After removing of circular osteophytes and patellar rim denervation, the patella surface is resected to restore is original thickness once the button is added. The patella thickness was measured with a caliper and a manual resection was performed restoring the original patella thickness. Care was taken to preserve at least 15 mm of patellar bone stock. Resection was verified intraoperatively using a Vernier caliper. The appropriate position for the patella button was chosen at the medial boarder of the patella to improve tracking. The uncovered bone area next to the component was left alone. The three holes for the pegs are drilled, and the appropriate button is added and cemented with Palacos^®^ (Heraeus Medical) bone cement. After that, patellar tracking was observed and the need for a lateral release was assessed.

The Genesis II patella buttons exist in different diameters: the 7.5 mm button is available in diameters of 26, 29, 32 and 35 mm, the 9 mm button in 38 and 41 mm, the design of the patella component is a symmetric all-polyethylene button.

In 946 cases the 7.5 mm Genesis II patella component (Smith & Nephew, Memphis, TN) and in 110 cases the 9 mm Genesis II patella component button was used, respectively.

The postoperative aftercare included weight-bearing as tolerated, and physiotherapeutic mobilization to receive an adequate range of motion. Patients were advised to avoid impact sports and heavy labor. A clinical and radiological follow-up consultation has been carried out after four weeks, three months and one year postoperatively. After the one-year follow-up, an X-ray and clinical consultation was conducted once a year. Loosening of patella components was determined on postoperative X-rays and confirmed on MRI. Loosening of patella buttons on MRI is accompanied by periprosthetic bone resorption at the implant–cement interface of the patella and synovial proliferations.

Statistical analysis was computed using SPSS® 26.0 (SPSS Inc. Chicago IL, USA). Results with *p* values < 0.05 were considered as statistically significant.

## Results

Of 1056 TKA, 35 cases (3.3%) showed aseptic loosening of the patella button (Table 1).

**Table 1:** Demographics

	Failure			No failure		
		Diameter			Diameter	
	Total	≤ 35 mm	≥ 38 mm	Total	≤ 35 mm	≥ 38 mm
n	35	24	11	1020	921	99
Age (years)	63.3 (41.6–81)	64.4 (50.1–81.1)	61.1 (41.3–77)	64.5 59.8 (23–95)	64.7 (23–95)	62.5 (40–85)
Male/female	20/15	9/15	11/0	322/698	229/692	93/6
BMI (kg/m^**2**^**)**	31.73 (21.1–47.9)	32.5 (21.1–47.9)	30.0 (24.1–36.3)	30.5 (17.7–59)	30.5 (17.7–59)	30.3 (18.4–50.2)
Time to revision (months)	52.5 (9–115)	61 (12–115)	34.2 (9–106)	N/A	N/A	N/A

**Fig. 1:**
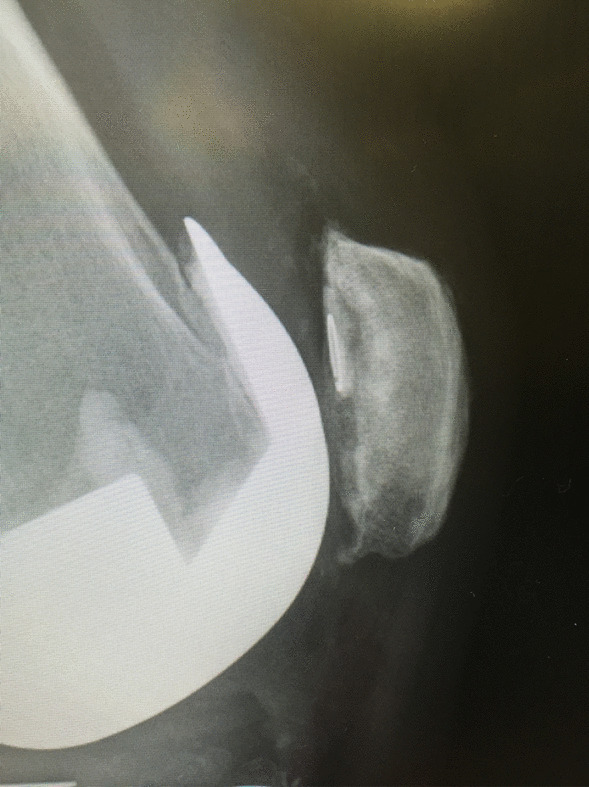
Patella loosening on preoperative radiographs of a 55-year-old male patient undergoing patella revision

**Fig. 2:**
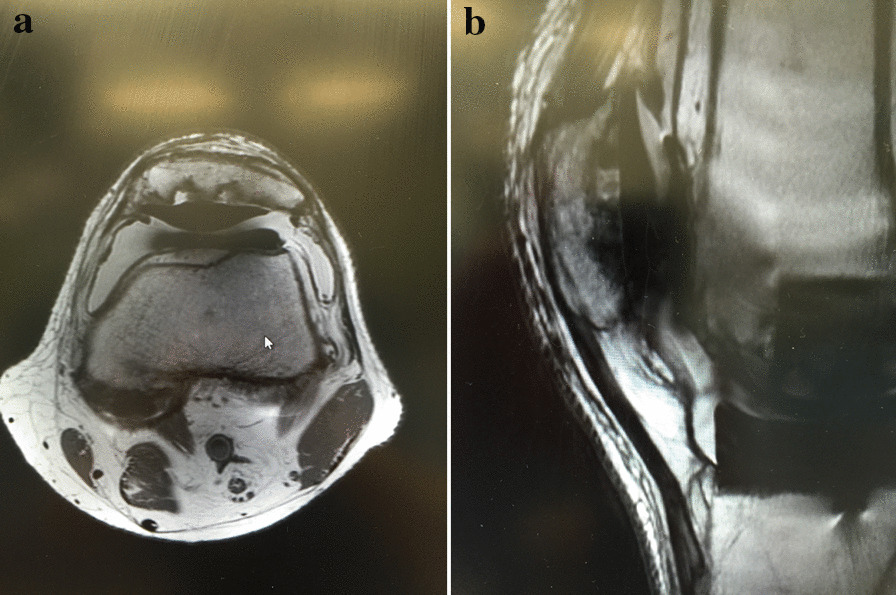
**a** Patella loosening on preoperative MRI transversal images of a 55-year-old male patient undergoing patella revision. **b** Sagittal MRI images of the same patient

**Fig. 3:**
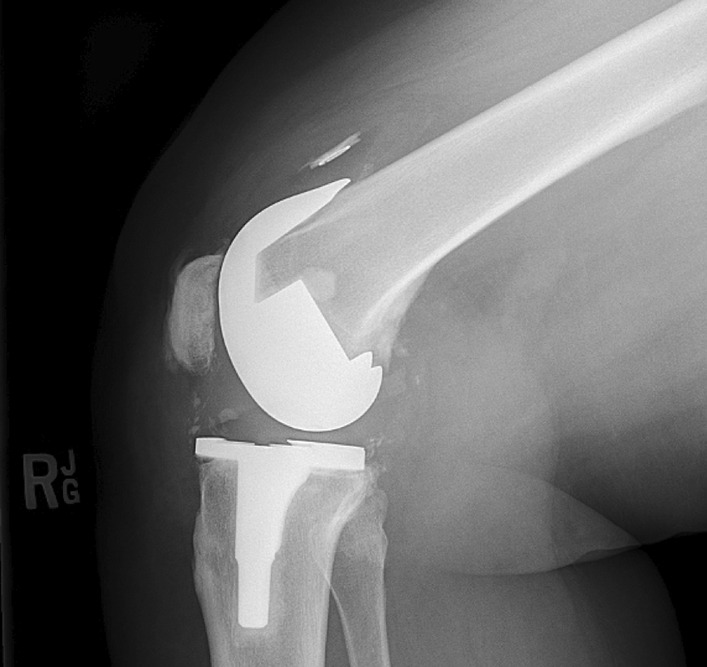
Superior migration of the button in a 56-year old female patient

11 occurred with the use of the 38 and 41 mm button (10%) and 24 (2.5%) with the use of the 26 to 35 mm button. The 9 mm thick 38 and 41 mm diameter button showed a significantly higher failure rate than the 7.5 mm button (*p* value: 0.00004). All patients underwent revision surgery. X-Rays were obtained for all patients and MRI in 34 patients (97.1%). Symptoms for patella loosening included pain in all patients and effusion in 82.9% of patients, radiographic signs for loosening of the patella button were osseous resorption of the patella in 62.9% and broken metal ring or migration in 8.6% (Table 2; Figs. 1, 2a, b, 3). Osteonecrosis of the patella was not present in our study due to the lack of isointense signals on T1 weighted images and intermediate signal intensity on T2 [19]. The rotation of the femoral and tibial components was evaluated on preoperative MRI. Average BMI of the patients with failure was 31.7 kg/m^2^ (range 21.1–47.9 kg/m^2^).

**Table 2:** MRI findings and symptoms at time of revision

Symptoms and findings	*N*	Percentage
Clinical signs		
Pain	35	100
Knee joint effusion	29	82.9
Instability	3	8.6
Decreased flexion	1	2.9
Radiographic findings		
Osteolysis	35	100
Osseous resorption	22	62.9
Fibrous membrane around patella button	21	60
Patella not centralized	7	20
Fracture	3 (2 only visible on MRI)	8.6
Broken metal ring/migration of button	3	8.6

Time to revision was median 52.5 months (range 9 to 106 months). Average age at revision was 63.3 years (range 41.3–81.1 years). Patients undergoing revision surgery did not have a lateral release during their primary procedure.

Indication for revision of the patella component was aseptic loosening in 34 patients (97.1%).

One female patient (age 71 years) suffered a patella fracture which was first treated nonoperatively. After secondary widening of the fracture gaps consecutive aseptic loosening of the patella component was diagnosed. The loose fragment was removed and four sutures were placed in the quadriceps tendon using the Krackow technique. Sutures were afterwards passed through drill holes in the patella and a reattachment of the quadriceps tendon was achieved by tying the threads at the distal patella pole. Finally, a 29 mm Genesis II patella button was cemented on to the larger remaining patella fragment.

Of the 34 cases with aseptic loosening of the button, 33 (97%) were treated with exchange of the patella button. In 20 cases a Journey II patella resurfacing button (Smith&Nephew) was cemented at time of revision surgery. An exchange to a cemented Genesis II biconvex inlay patella button was performed in 13 patients. For the revision surgery, a 35 mm button was used in 18 patients, a 32 mm button in four patients, a 41 mm button in three patients, a 38 mm button in three patients, a 29 mm button in two patients, a 26 mm button in two patients, and a 23 mm button in one patient. Patella thickness ranged from 15.6 to 24.5 mm (average 18.5 mm) preoperatively, before revision surgery from 12.3 to 20.1 mm (average 16.0 mm) and after revision surgery from 9.3 to 17.6 mm (average 14.0 mm), measured without patella button.

An additional insert exchange to a larger insert was performed in four patients due to slight laxity in flexion and extension during revision surgery. One patient intraoperatively showed a disruption of the anterior cement mantle under the tibia, so a revision of the tibial and the femoral components was performed with a Legion Revision implant (Smith and Nephew, Memphis TN) and a constrained insert.

Two patients with significant loss of patellar bone stock were treated with bone grafting of the patella alone. In one of those patients an inlay exchange to a higher inlay was performed due to increased laxity on clinical exam.

Mean follow-up after revision surgery was 50.4 months (range 11–130 months).

No further surgeries were necessary and no complications occurred in the follow-up period.

## Discussion

The current study reports an aseptic failure rate of the Genesis II patella button of 3.3% predominantly as a result of the higher failure rate (10%) with the use of the 38 and 41 mm diameter patella components in the current cohort of 1056 patients. Aseptic loosening occurred in 3.1% of patients in our cohort. Careful screening of the integrity of the metal marker ring, the position of the button and the interface between bone and cement is recommended to detect this failure mode. Heyse et al. reported that MRI is adequate to diagnose implant loosening in patients with radiographic suspicion for loosening and can also be used to determine the rotation of the femoral and tibial component [20, 21].

Aseptic loosening rates of patella buttons in the literature have been described up to 4.8–9.8% [12, 14, 22]. The 10-year survival of biconvex patella buttons was 97% for aseptic loosening in 521 TKA with patella implants [23]. BMI over 30 kg/m^2^ is associated with patella loosening in the literature [12, 13] and was also a risk factor for loosening in the current study. Rheumatoid arthritis was also proven to be a risk factor for patella-related complications [24]. Similar to our study, men were more frequently affected by loosening of the patella compared to women [25].

The principles of patellofemoral biomechanics have been described by Schindler [26]. Dome shaped patella buttons, similar to the Genesis II button, provide congruency in flexion up to 70°. At flexion over 70° the patella is exposed to higher eccentric pressure forces.

The idea of larger patella designs to cover a larger area of the patella as well as the use of thinner patella components might provide less resistance to eccentric forces acting on the button. Thinner and larger buttons might prematurely loosen due to higher shear forces on the edges of the button.

Increasing patella thickness influences patellofemoral pressure in TKA and the pressure was greatest at 90 degrees of knee flexion, and a two mm increase or decrease resulted in a 20% increase or decrease of patellofemoral pressure [27]. The increased patellofemoral pressure in thicker components could also have an influence on the loosening rate of those buttons.

Jhurani et al. [28] found no loosening with the use of a three-pegged, cross-linked polyethylene 6.2 mm patella button in patients with thinner patellar bone stock of maximal 20 mm over a follow-up period of average 26.72 months. This suggests that beside thickness overall diameter of the button might have to be taken into consideration with the current study showing higher failure rates in 38 and 41 mm buttons regardless of their 9 mm thickness.

In a study by Ritter et al. patella loosening occurred in 3.7% of patients, who have had a lateral release during their primary procedure and in 1.8% of patients that did not have a lateral release [25].

Avascular necrosis including resorption of the patella is a rare condition and has been described in different case reports [29, 30] after TKA and arthroscopy. Nakagawa et al. [31] recommend preserving continuity of the retinaculum and extensor mechanism, to avoid patellar osteonecrosis. A cadaveric study of De Bell et al. shows that blood supply occurs from the superior and inferior genicular arteries to the lateral patella and during lateral release, those arteries are at risk and severance can lead to avascular necrosis of the patella [32]. We could not identify patients with avascular necrosis in our cohort.

Another goal in patella resurfacing during TKA is to avoid overstuffing, proper patella tracking and restore the preoperative patella thickness [33, 34] to minimize the risk of a decrease of postoperative knee flexion, which could also be achieved by thin buttons.

The use of a patella button is considered a standard of care in the United States and at the senior author’s institution [2, 3], however, some studies detected no difference in clinical outcome and survivorship after TKA with and without patellar resurfacing [24, 35]. Feng et al. found at a minimum follow-up of ten years no significantly difference using scores, but the incidence of anterior knee pain was non-significantly higher in the non-resurfacing group [24]. According to Hwang et al., there was no significant difference in bilateral TKA with only one side resurfaced [36]. In our cohort bilateral loosening was present in five patients needing revision surgery on both sides.

The current study has following major limitations: (1) we did not have a control group with another implant; (2) the retrospective character of the study; (3) the average follow-up period of 50.4 months is relatively short, however, patients were operated on more than five years ago and it is assumed that patients with symptoms would have presented back to the authors’ institution; (4) patella thickness was measured at the center of the patella and more peripheral areas might have had larger bone defects; (5) taller and male patients might require larger components and these larger components might be therefore exposed to increased loads compared to smaller components increasing their risk of failure.

## Conclusion

The current study reports a 10% aseptic loosening rate of the 38 mm and 41 mm patella button. The combination of increased shear forces due to the larger diameter and the relatively small peg size might explain the increased failure rate. The current study does not recommend the use of 38 mm and larger diameter patella components. Careful screening of postoperative radiographs can help to identify patients with aseptic loosening and MR-imaging is the recommended imaging tool to confirm the diagnosis.

## Data Availability

Not applicable.
